# Editorial: Magnetic particle-assisted sensing and magnetic biosensors

**DOI:** 10.3389/fbioe.2024.1518156

**Published:** 2024-11-11

**Authors:** Dongfei Chen, Teresa Zardán Gómez de la Torre, Fuxiang Wei, Bo Tian, Kai Wu

**Affiliations:** ^1^ Graduate School of Biomedical Engineering, The University of New South Wales, Sydney, NSW, Australia; ^2^ Department of Material Sciences and Engineering, Ångström Laboratory, Uppsala University, Uppsala, Sweden; ^3^ College of Life Science and Technology, Huazhong University of Science and Technology, Wuhan, China; ^4^ School of Basic Medical Sciences, Central South University, Changsha, China; ^5^ Department of Electrical and Computer Engineering, Texas Tech University, Lubbock, TX, United States

**Keywords:** magnetic particles, magnetic biosensors, magnetoresistive sensors, magnetic manipulations, point-of-care tests

Advances in nanoscience have driven substantial progress in biomedical sensing technologies, positioning magnetic nanoparticles (MNPs) as critical functional elements in modern sensing platforms. MNPs are particularly advantageous in biomedical applications due to their biocompatibility, stability, and unique ability to be manipulated non-invasively via external magnetic fields. These features enable MNPs to act as precise and versatile tools within analytical systems, facilitating high sensitivity and selectivity even in complex biological matrices. Importantly, MNPs exhibit strong contrast against biological materials, allowing the detection of trace biomolecules—a key requirement in early-stage diagnostics. As a result, there has been remarkable growth in the application of magnetic particle-assisted sensing and magnetic biosensors over the past decade.

Magnetic particle-assisted sensing leverages the ability to apply external magnetic fields (static or dynamic) to MNPs, enabling remote manipulation of the particles in a diverse environment (Gloag et al., 2019). Typically, these fields provide precise control over MNP functions such as extraction, stirring, and sorting, while also facilitating specific biophysical measurements via magnetic, optical, or electrochemical signal transduction. Additionally, magnetic forces can enhance particle interactions, overcoming diffusion limits to improve reaction kinetics for fast and uniform assay performance (Xiao et al., 2022). This capability is essential in applications requiring rapid response times, including point-of-care diagnostics and environmental monitoring.

Despite these advantages, several challenges persist in the use of MNPs for sensing. For example, magnetic incubation processes can lead to non-specific binding, which increases background signals and subsequently reduces system sensitivity. Achieving multiplex detection—where multiple targets are identified simultaneously—adds further complexities, as distinguishing between signals from various types of magnetic particles remains challenging. Furthermore, current techniques such as magnetic relaxation switching often face difficulties in differentiating signals when multiple particle types are present. Overcoming these limitations is critical to advancing MNP-enabled sensing technologies, necessitating innovations in materials fabrication, signal processing, and sensor design to enhance system robustness and versatility (Wu et al., 2019).

This Research Topic in *Frontiers in Bioengineering and Biotechnology* features a curated selection of pioneering research on magnetic particle-assisted sensing and biosensing technologies. The articles introduce several innovative solutions to current limitations, expanding the practical applicability of MNPs across maternal health, infectious disease diagnostics, environmental monitoring, and multiplexed biomarker analysis fields.

Sveiven et al. introduce advancements in MNP-based biosensors aimed at enhancing signal-to-noise ratios for cancer diagnostics, thus reducing false positives and improving the precision of early-stage cancer detection. By harnessing magnetic signal transduction, these biosensors offer rapid detection and are highly sensitive to small biomolecular changes in the early phases of cancer progression, providing promising avenues for clinical use in early intervention.

Xu et al. investigate the application of MNPs in infectious disease diagnostics, demonstrating significant accuracy in pathogen detection even at low sample volumes. Acting as effective binding platforms, MNPs enhance target capture and enable precise pathogen identification, underscoring their value in resource-limited settings where rapid, accurate detection can have a substantial public health impact.

In environmental monitoring, Camacho et al. showcase MNPs’ potential in detecting pollutants in water, illustrating how magnetic particle-based assays enable high-throughput analysis while reducing cross-reactivity. Through specialized catalytic surfaces, this research introduces scalable methods for pollutant detection, essential for maintaining environmental safety and supporting real-time water quality monitoring.

Sveiven et al. highlight MNPs’ role in multi-parameter assays, where they enable simultaneous detection of multiple biomarkers. This multiplexing capability enhances diagnostic precision, supporting complex, multi-analyte tests necessary for personalized healthcare and advanced disease monitoring. Such applications emphasize the transformative potential of MNPs in diagnostics by providing a comprehensive view of biomolecular landscapes in real time.

In summary, the articles in this Research Topic mark substantial advancements in magnetic particle-assisted sensing and biosensing. They demonstrate the transformative potential of MNP-based technologies to address critical gaps in biomedical, environmental, and diagnostic applications. By tackling challenges such as non-specific interactions, complex multiplexing, and signal differentiation, MNPs offer a pathway toward enhanced sensitivity, speed, and specificity in next-generation sensing technologies. Beyond showcasing these innovations, this issue aims to inspire ongoing research to optimize MNP functionalization, refine signal processing, and advance assay design, supporting the evolving needs of healthcare, environmental science, and personalized medicine.
